# Establishment and Validation of a Ferroptosis-Related lncRNA Signature for Prognosis Prediction in Lower-Grade Glioma

**DOI:** 10.3389/fneur.2022.861438

**Published:** 2022-06-27

**Authors:** Qian-Rong Huang, Jian-Wen Li, Ping Yan, Qian Jiang, Fang-Zhou Guo, Yin-Nong Zhao, Li-Gen Mo

**Affiliations:** ^1^Department of Neurosurgery, Guangxi Medical University Cancer Hospital, Nanning, China; ^2^Department of Hepatobiliary Surgery, Guangxi Medical University Cancer Hospital, Nanning, China

**Keywords:** lower-grade glioma, ferroptosis, lncRNA, signature, prognosis

## Abstract

**Background:**

The prognosis of lower-grade glioma (LGG) is highly variable, and more accurate predictors are still needed. The aim of our study was to explore the prognostic value of ferroptosis-related long non-coding RNAs (lncRNAs) in LGG and to develop a novel risk signature for predicting survival with LGG.

**Methods:**

We first integrated multiple datasets to screen for prognostic ferroptosis-related lncRNAs in LGG. A least absolute shrinkage and selection operator (LASSO) analysis was then utilized to develop a risk signature for prognostic prediction. Based on the results of multivariate Cox analysis, a prognostic nomogram model for LGG was constructed. Finally, functional enrichment analysis, single-sample gene set enrichment analysis (ssGSEA), immunity, and m6A correlation analyses were conducted to explore the possible mechanisms by which these ferroptosis-related lncRNAs affect survival with LGG.

**Results:**

A total of 11 ferroptosis-related lncRNAs related to the prognosis of LGG were identified. Based on prognostic lncRNAs, a risk signature consisting of 8 lncRNAs was constructed and demonstrated good predictive performance in both the training and validation cohorts. Correlation analysis suggested that the risk signature was closely linked to clinical features. The nomogram model we constructed by combining the risk signature and clinical parameters proved to be more accurate in predicting the prognosis of LGG. In addition, there were differences in the levels of immune cell infiltration, immune-related functions, immune checkpoints, and m6A-related gene expression between the high- and low-risk groups.

**Conclusion:**

In summary, our ferroptosis-related lncRNA signature exhibits good performance in predicting the prognosis of LGG. This study may provide useful insight into the treatment of LGG.

## Introduction

Gliomas are common and deadly primary tumors of the central nervous system, accounting for nearly 80% of all primary malignant brain tumors (1). Gliomas are classified by the World Health Organization (WHO) as grades I–IV based on their histological type and malignant behavior (2). Diffuse grades II–III gliomas are commonly defined as lower-grade gliomas (LGGs), which are characterized by large differences in biological and clinical behavior (3). The survival time of patients with LGG is highly variable, ranging from 1 to 15 years, with some patients being very sensitive to treatment, while others rapidly develop into highly malignant glioblastoma (GBM, grade IV) (4, 5). Although molecular diagnosis has been incorporated into the classification of LGG (6), the prognosis of patients cannot be accurately predicted by existing methods. Hence, it is necessary to further explore the prognostic markers of LGG, which is also conducive to the discovery of potential therapeutic targets.

Of note, more and more pieces of evidence have suggested that ferroptosis is linked to the prognosis of cancer patients, such as those with pancreatic ductal carcinoma, hepatocellular cancer, and renal clear cell carcinoma (7–9). Ferroptosis is a form of iron-dependent regulated cell death caused by excessive lipid peroxidation, which has been involved in the occurrence and progression of various types of diseases (10, 11). Studies have shown that ferroptosis also has a tumor suppressor function that could be used for cancer treatment (12–14). The long non-coding RNAs (lncRNAs) are defined as a subset of RNA molecules with ~200 nucleotides (15, 16). They not only participate in gene regulation (17) but also involve tumor biological behavior, such as occurrence, development, and metastasis (18, 19). In addition, there is evidence that abnormal regulation of specific lncRNAs is associated with the ferroptosis process in colorectal cancer and leukemia (20, 21). However, these specific lncRNAs and their prognostic values are still rarely explored in LGG.

Here, we aimed to investigate the prognostic role of ferroptosis-related lncRNAs in LGG and develop a novel risk signature for survival prediction. We first integrated multiple datasets to screen for prognostic ferroptosis-related lncRNAs. A risk signature for prognostic prediction of LGG was then constructed by least absolute shrinkage and selection operator (LASSO) regression analysis. In addition, a nomogram was established to predict the 1-, 3-, and 5-year survival rates by combining this signature with other independent prognostic parameters. Eventually, we investigated the relationship of the risk signature with underlying biological functions, immune functions, and m6A-related genes.

## Materials and Methods

### Data of Patients With LGG in This Study

Three independent LGG cohorts were enrolled in the present study. Expression profiles (RNA-seq) and related clinical data were collected from The Cancer Genome Atlas (TCGA, https://portal.gdc.cancer.gov/), Chinese Glioma Genome Atlas (CGGA, http://www.cgga.org.cn/), and Gravendeel (http://gliovis.bioinfo.cnio.es/) databases. A total of 259 ferroptosis-related genes were downloaded from the FerrDb database (http://www.zhounan.org/ferrdb/) (22). Pearson analysis was then utilized to evaluate the association between ferroptosis-related genes and lncRNAs in LGG. In this study, lncRNAs with correlation coefficients |R| > 0.4 and *p* < 0.001 were selected as ferroptosis-related lncRNAs. To screen for prognostic ferroptosis-related lncRNAs, we included patients with available survival information and overall survival (OS) ≥30 days. Finally, 408 patients from the TCGA dataset were included in the training cohort, while 590 patients from the CGGA dataset and 104 patients from the Gravendeel dataset served as the validation cohort (Table 1).

**Table 1:** Clinicopathological information of patients with lower-grade glioma (LGG) in the training and validation cohorts.

**Clinicopathological features**		**Training cohort**	**Validation cohorts**	
		**TCGA** **(*n* = 408)**	**CGGA** **(*n* = 590)**	**Gravendeel** **(*n* = 104)**
Age (years)	≤40	195	306	40
	>40	213	283	64
	NA	0	1	0
Gender	Male	226	340	68
	Female	182	250	36
Grade	II	196	269	23
	III	212	321	81
IDH status	Mutant	331	413	46
	Wild-type	75	138	38
	NA	2	39	20
1p19q codeletion status	Non-codeletion	270	370	NA
	Codeletion	138	180	NA
	NA	0	40	NA
MGMT promoter status	Methylated	NA	284	NA
	Un-methylated	NA	199	NA
	NA	NA	107	NA
PR type	Primary	NA	408	NA
	Recurrent	NA	182	NA

*IDH, isocitrate dehydrogenase; MGMT, O-6-methylguanine-DNA methyltransferase*.

### Development of a Risk Signature

Univariate Cox regression analysis was conducted to screen out prognostic lncRNAs (*p* < 0.05) in these three cohorts. To ensure accuracy, overlapping prognostic lncRNAs from the three cohorts were extracted as candidate lncRNAs. Then, LASSO regression analysis was used in the training cohort to further narrow the range of candidate lncRNAs and establish a risk signature. The risk score formula was as follows: risk score = Σ explncRNAi × βi (where explncRNAi represents the expression of the selected lncRNA and βi represents the corresponding coefficient). In this study, the median value of the risk score was used as the cutoff value for the high- and low-risk groups. Based on the expression values of the lncRNAs included in the signature, principal component analysis (PCA) was carried out *via* the “scatterplot3d” package in R to assess potential differences between the two subgroups. The Kaplan–Meier method was utilized to measure the difference in OS between the high- and low-risk subgroups. The time-dependent receiver operating characteristic (tROC) curve was then plotted using the “timeROC” package to evaluate the predictive accuracy of the risk signature. These assessment methods were also performed in the CGGA and Gravendeel cohorts to validate their predictive value. In addition, we analyzed the relationship between the risk score and clinical characteristics of LGG.

### Development of a Prognostic Nomogram

Univariate and multivariate Cox regression analyses were further carried out to determine the independent prognostic value of the risk signature in LGG. Next, we incorporated independent prognostic parameters of the training cohort to establish a nomogram, which was carried out through the “rms” package. The prediction performance of the nomogram was evaluated through the tROC curve, decision curve analysis (DCA), calibration curve, Kaplan–Meier method, and concordance index (C-index).

### Functional Enrichment Analyses

To investigate the related biological roles of the risk signature, we screened out differentially expressed genes (DEGs, |log_2_FC| > 1 and false discovery rate [FDR] < 0.05) between the two subgroups in TCGA cohort using the “limma” package. Based on DEGs, Gene Ontology (GO) analysis and Kyoto Encyclopedia of Genes and Genomes (KEGG) enrichment analysis were carried out through the “clusterProfiler” package, and annotation results reaching *p* < 0.05 were considered to be significant.

### Correlation Analysis of the Risk Signature With Immunity and m6A

We first compared differences in the levels of immune responses in the two risk subgroups using several algorithms (TIMER, CIBERSORT, CIBERSORT-ABS, QUANTISEQ, MCPCOUNTER, XCELL, and EPIC) (23). Scores of 16 types of immune cell infiltration and activity of 13 immune-related functions were also compared using single-sample gene set enrichment analysis (ssGSEA), which was performed *via* the “gsva” package. In addition, we analyzed the correlation of the lncRNA signature with potential immune checkpoints and m6A-related genes.

### Statistical Analysis

The continuous variable (risk score) between two groups was compared using Student's *t*-test, and the chi-square test was used for categorical variables. Comparisons of immune cells, immune functions, immune checkpoints, and m6A-related gene expression between the two risk groups were conducted using the Wilcoxon test. The difference in OS of patients with LGG between groups was measured by Kaplan–Meier curves and log-rank tests. The remaining statistical methods are described above. A value of *p* < 0.05 was considered statistically significant. Statistical analysis and graph production of this study were completed by R software (v3.6.3).

## Results

### Identification of Prognostic lncRNAs for LGG

**Figure 1:**
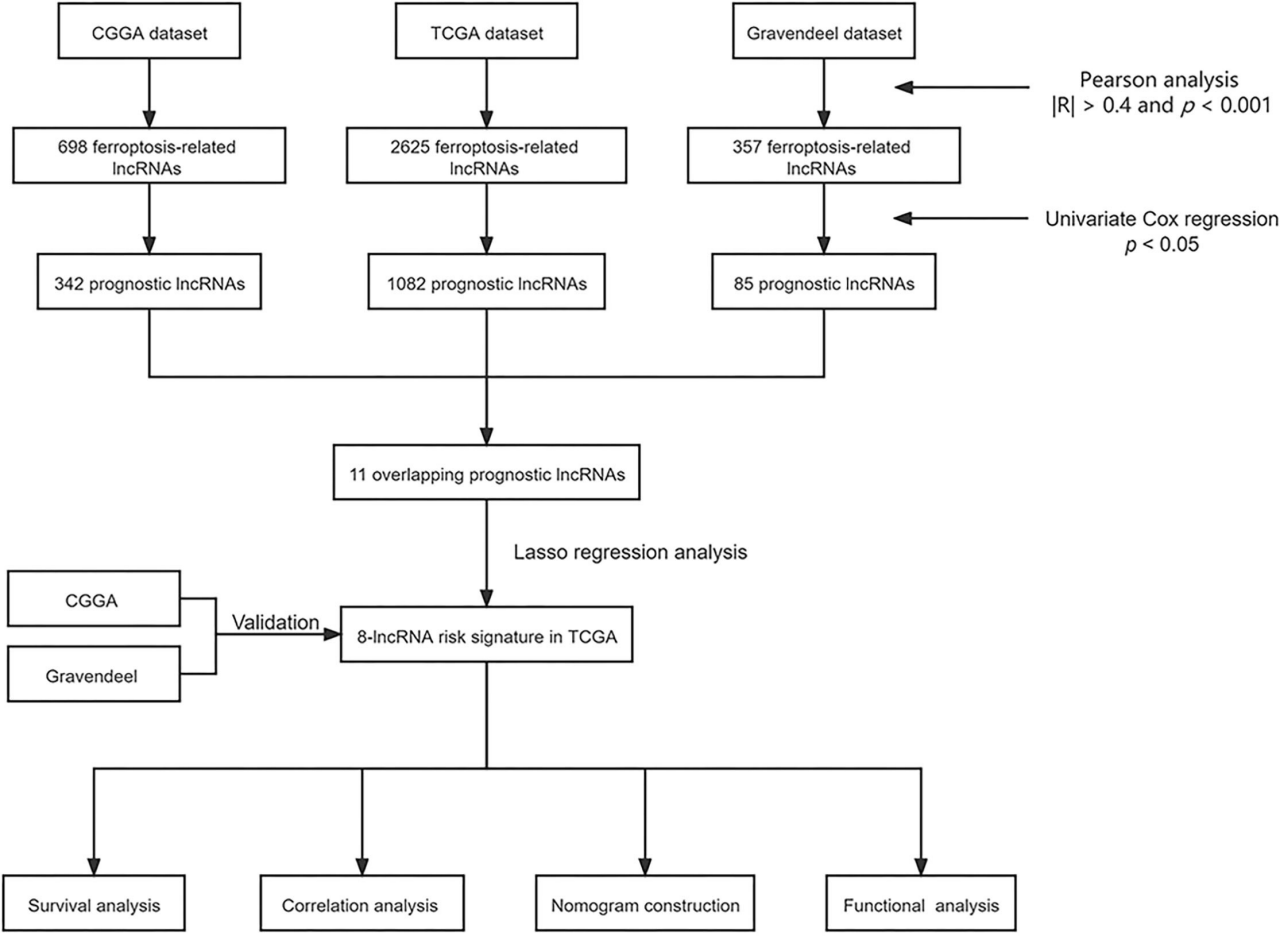
The flow chart of our study.

**Figure 2:**
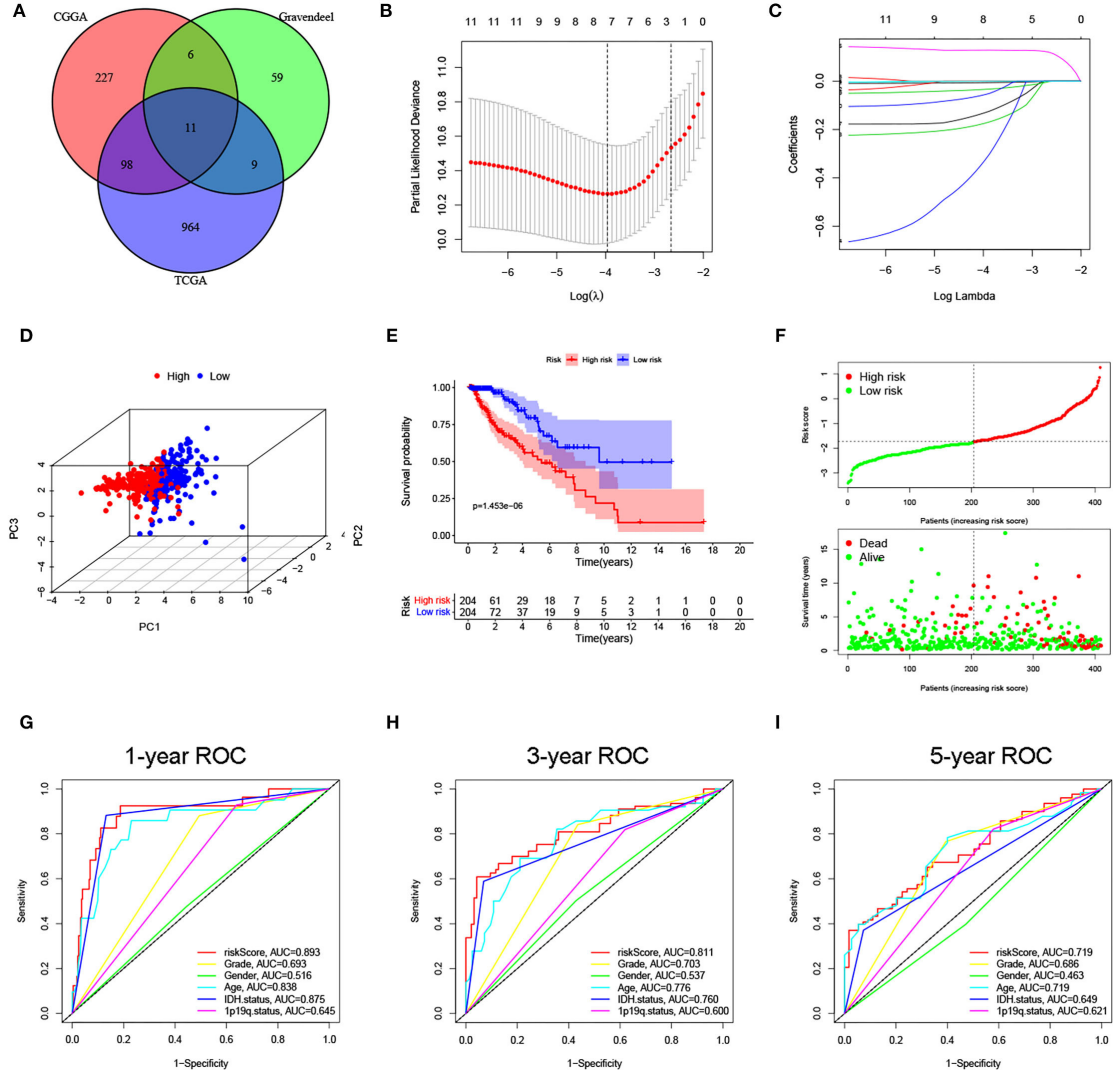
Construction of a ferroptosis-related long noncoding RNAs (lncRNA) signature for lower-grade glioma (LGG) in The Cancer Genome Atlas (TCGA) cohort. **(A)** Overlapping prognostic lncRNAs in 3 cohorts were screened out. **(B,C)** Minimum criteria and coefficients were calculated by least absolute shrinkage and selection operator (LASSO) analysis. **(D)** The distribution difference is shown between the two subgroups by PCA. **(E)** Kaplan–Meier curve of the two subgroups. **(F)** The distribution plot of risk score, survival status, and overall survival (OS) time. **(G–I)** area under the curve (AUC) values of 1-, 3-, and 5-year OS for the risk score and clinical features.

The flow chart of our study is shown in Figure 1. By Pearson correlation analysis, we obtained 2,625, 698, and 357 ferroptosis-related lncRNAs from TCGA, CGGA, and Gravendeel datasets, respectively. Among them, the numbers of prognostic lncRNAs (*p* < 0.05) in TCGA, CGGA, and Gravendeel cohorts were 1,082, 342, and 85, respectively. Ultimately, we found that 11 prognostic lncRNAs overlapped in all three cohorts, and these 11 lncRNAs were selected for subsequent analysis (Figure 2A).

### Construction and Validation of the Risk Signature

Based on prognostic lncRNAs, an 8-lncRNA prognostic signature was constructed through LASSO regression analysis in the TCGA cohort (Figures 2B,C). The risk score formula was as follows: risk score = expressionLINC00844 × (−0.0064) + expressionFAM66C × (−0.1737) + expressionTUBA3FP × (−0.3259) + expressionSNHG8 × (−0.0002) + expressionCRNDE × (0.1282) + expressionHAR1A × (−0.1302) + expressionLINC00641 × (−0.0337) + expressionMYCNOS × (−0.0555). We then divided patients with LGG into high- and low-risk subgroups according to the median risk score. PCA showed stable and significant differences in distribution between the two risk subgroups (Figure 2D). The Kaplan–Meier curve suggested that patients in the high-risk group had a shorter OS time than those in the low-risk group (*p* < 0.001; Figure 2E). As seen from the distribution plot, the number of surviving cases and survival time was decreased in the high-risk group when compared with the low-risk group (Figure 2F). The area under the curve (AUC) of the tROC confirmed that the signature was a good predictor of survival and was superior to traditional clinicopathological features (Figures 2G–I).

**Figure 3:**
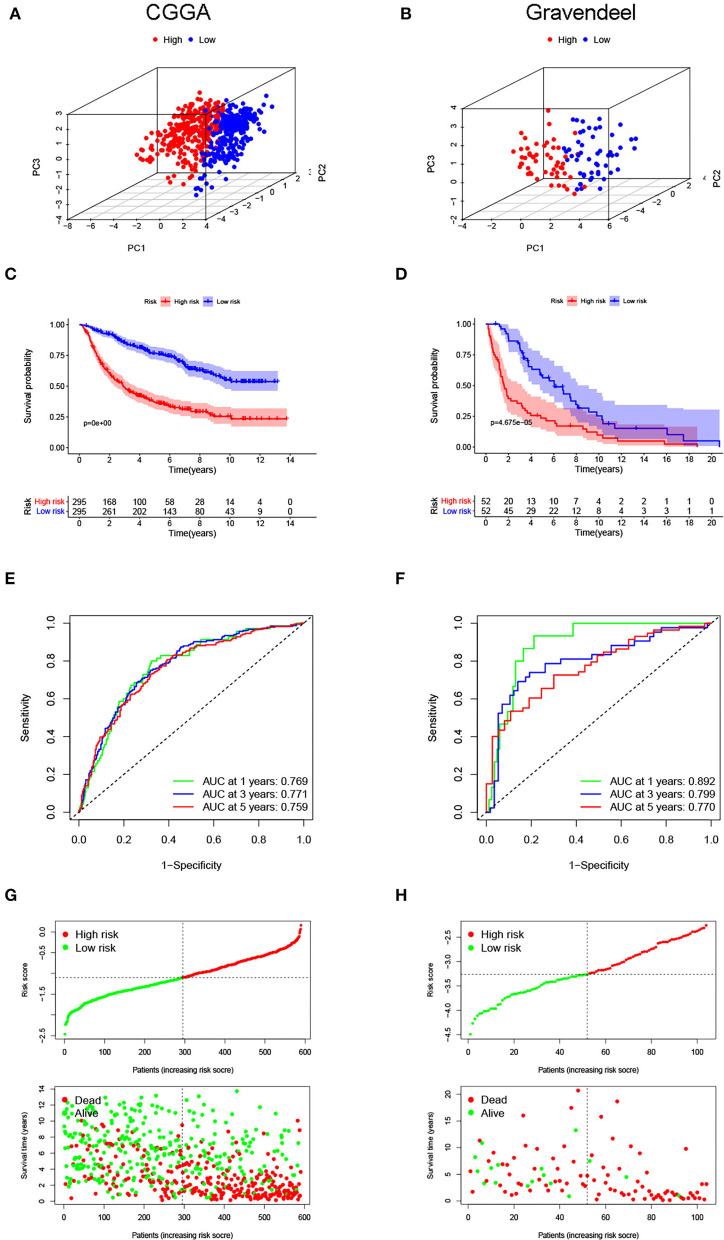
Evaluated predictive value of the risk signature in the validation cohorts. Principal component analyses (PCAs) for the **(A)** Chinese Glioma Genome Atlas (CGGA) and **(B)** Gravendeel cohorts. **(C,D)** Kaplan–Meier curves for survival in the validation cohorts. **(E,F)** Time-dependent ROC curves are used to assess the prediction accuracy. **(G,H)** Distribution plots of the risk score, survival status, and survival time in the validation cohorts.

Next, the same analyses were carried out in the CGGA and Gravendeel cohorts for external validation. In both validation cohorts, PCA also showed obvious differences in the distribution between high- and low-risk subgroups (Figures 3A,B). Patients with higher risk scores had shorter OS times (*p* < 0.001), which was consistent with the findings of the TCGA cohort (Figures 3C,D). Similarly, AUC values for predicting OS at 1-, 3-, and 5-year were high in both cohorts, all >0.75 (Figures 3E,F). The same trend was observed in survival distribution plots, with shorter OS times and more deaths as the risk score increased (Figures 3G,H). All these results suggested that the risk signature could stably and accurately predict the prognosis of LGG.

### Relationship of the Risk Signature With LGG Features

**Figure 4:**
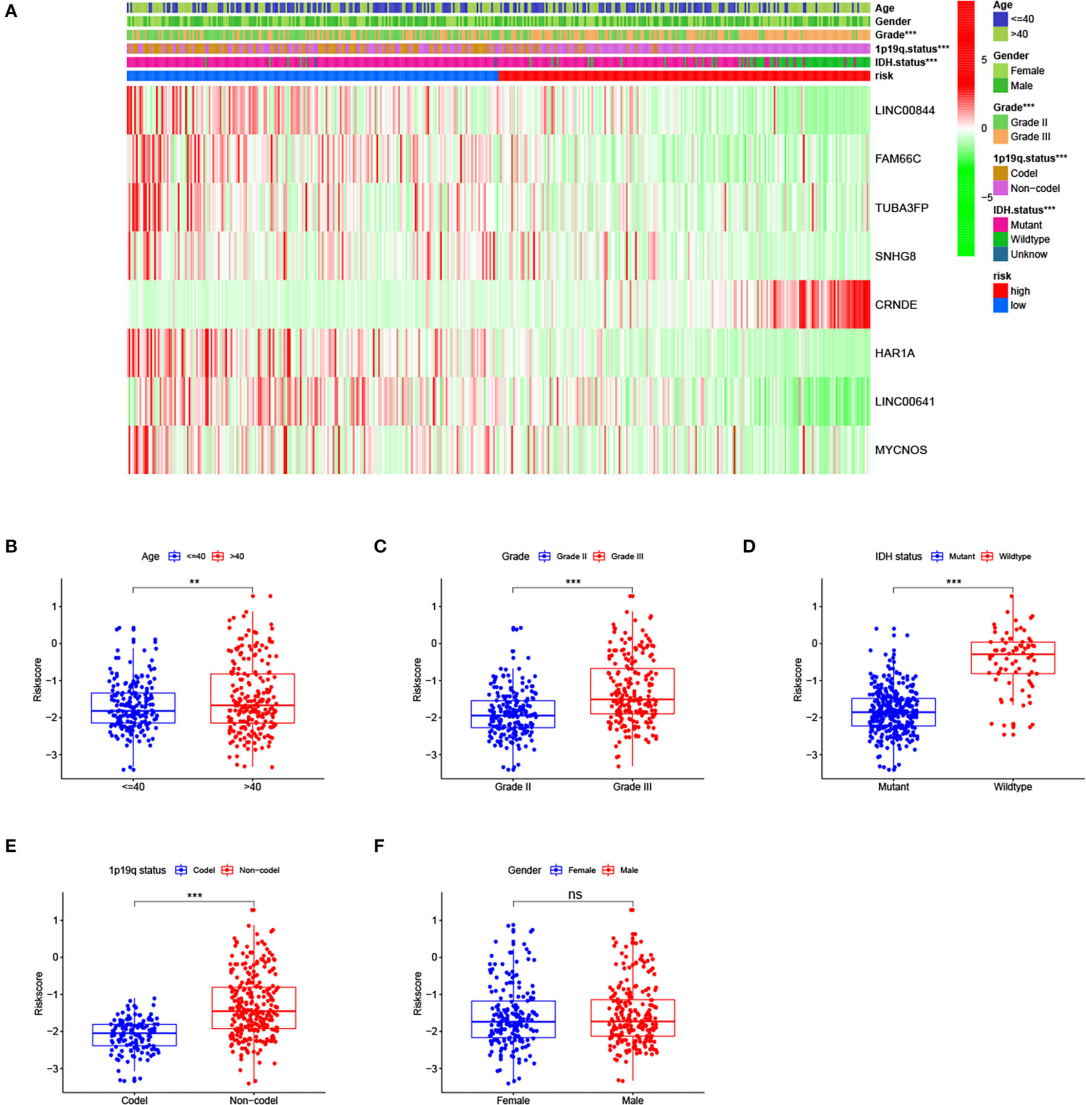
Relationship of the risk signature with clinical characteristics of lower-grade glioma (LGG) in The Cancer Genome Atlas (TCGA) cohort. **(A)** Heatmap for risk score, selected long noncoding RNAs (lncRNAs), and clinicopathological features. **(B–E)** LGG patients with age >40, grade III, isocitrate dehydrogenase (IDH) wild-type, and 1p19q non-codeletion had higher risk scores, and **(F)** there was no difference between men and women. ** *p* < 0.01, *** *p* < 0.001, ns, no significance.

As shown in Figure 4A, the proportion of patients with grade III, isocitrate dehydrogenase (IDH) wild-type, and 1p19q non-codeletion is higher in the high-risk group than in the low-risk group (*p* < 0.001). The heatmap shows the distribution of risk signature lncRNA expression. With the increase in the risk score, the expression level of the risk lncRNA (CRNDE) was increased, while the expression levels of the remaining protective lncRNAs were decreased. We also analyzed risk score levels in LGG classification based on clinical and molecular characteristics. We observed that patients with age > 40, grade III, IDH wild-type, and 1p19q non-codeletion had higher risk scores (Figures 4B–E), while there was no difference between male and female patients (Figure 4F). Overall, these results suggested that a high-risk score was closely linked to characteristics of high malignancy in LGG.

### Construction of a Prognostic Nomogram Model

**Figure 5:**
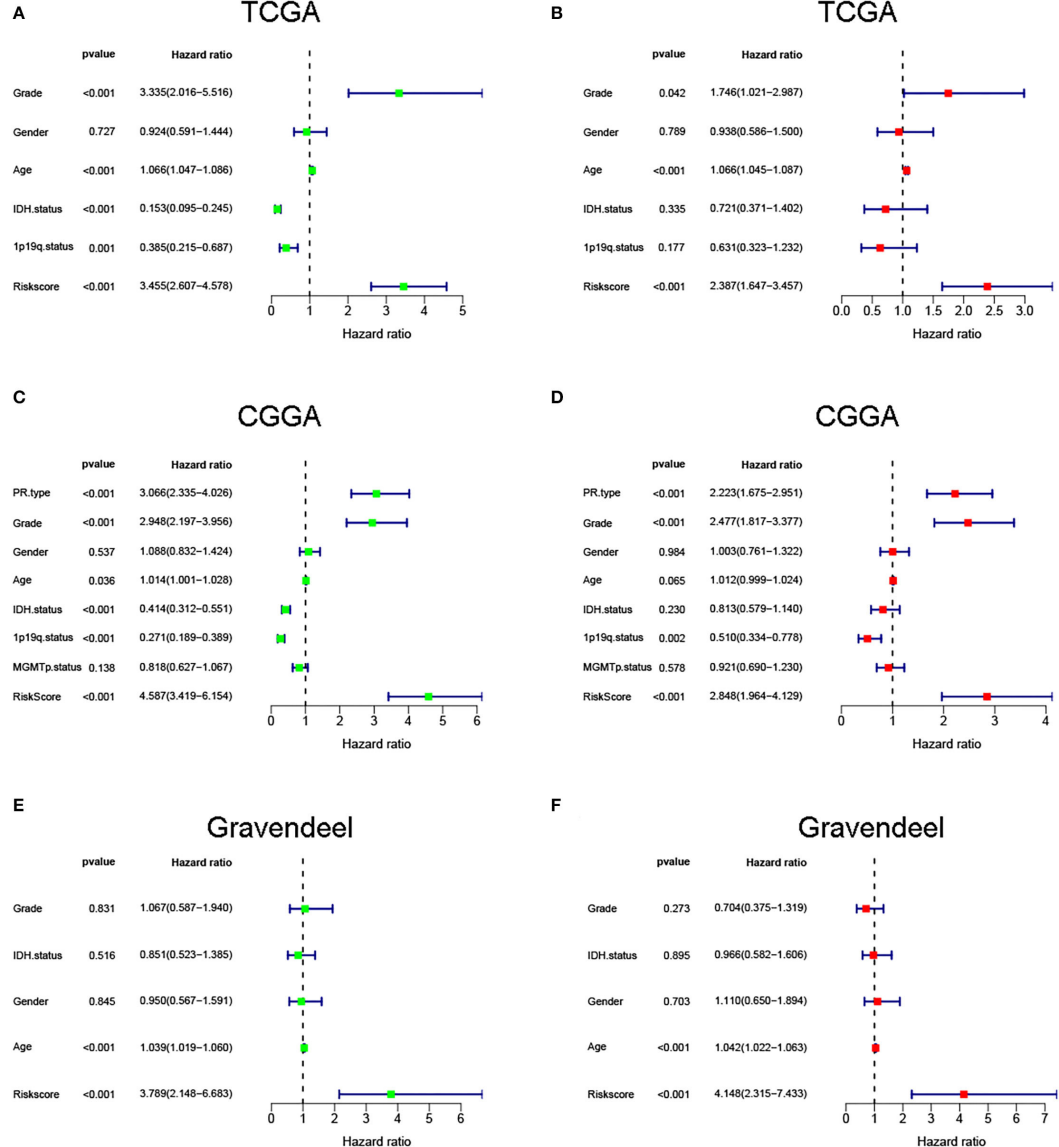
Univariate and multivariate regression analyses were performed in **(A,B)** The Cancer Genome Atlas (TCGA), **(C,D)** Chinese Glioma Genome Atlas (CGGA), and **(E,F)** Gravendeel cohorts.

**Figure 6:**
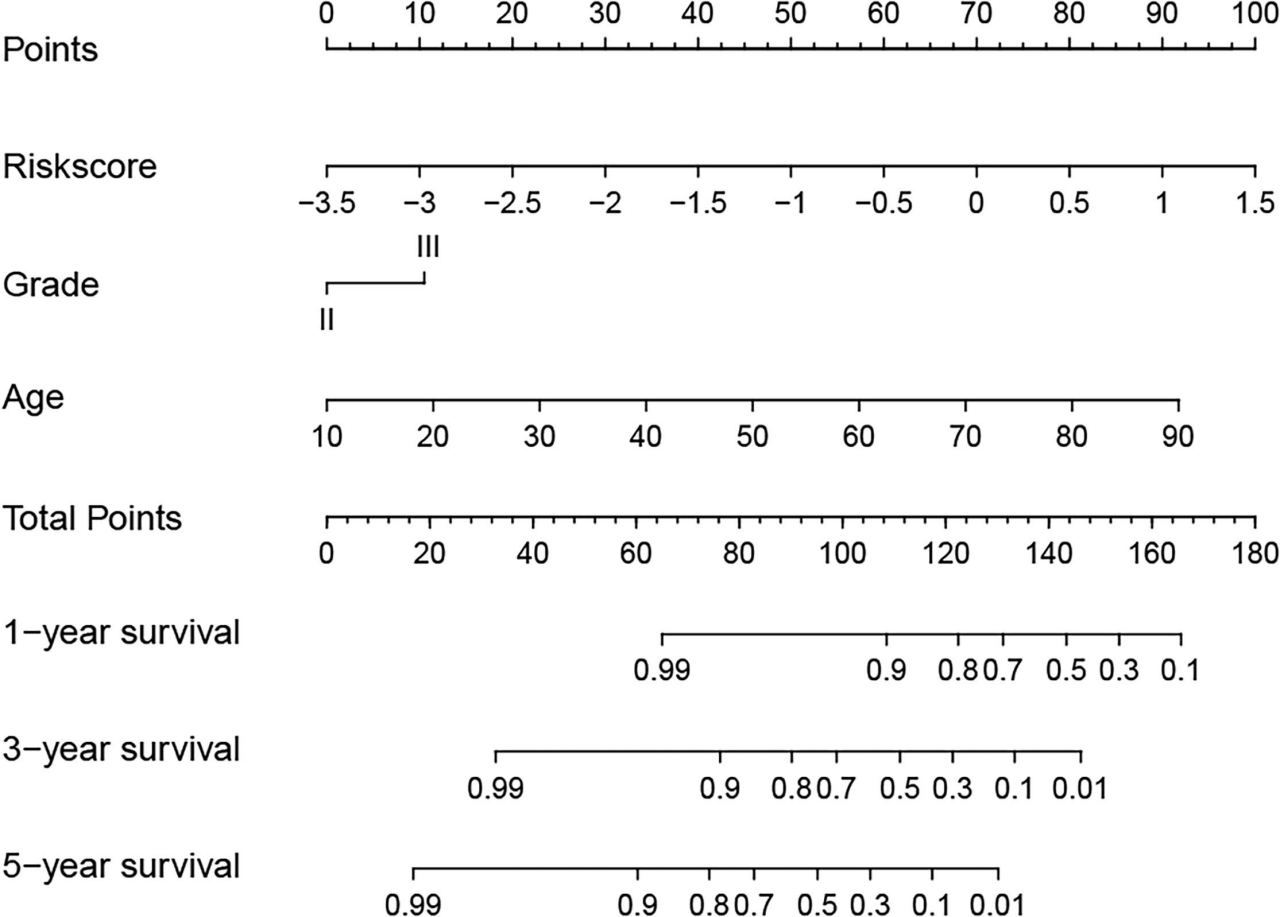
A nomogram model was established to predict the overall survival (OS) of lower-grade glioma (LGG) in The Cancer Genome Atlas (TCGA) training cohort.

**Figure 7:**
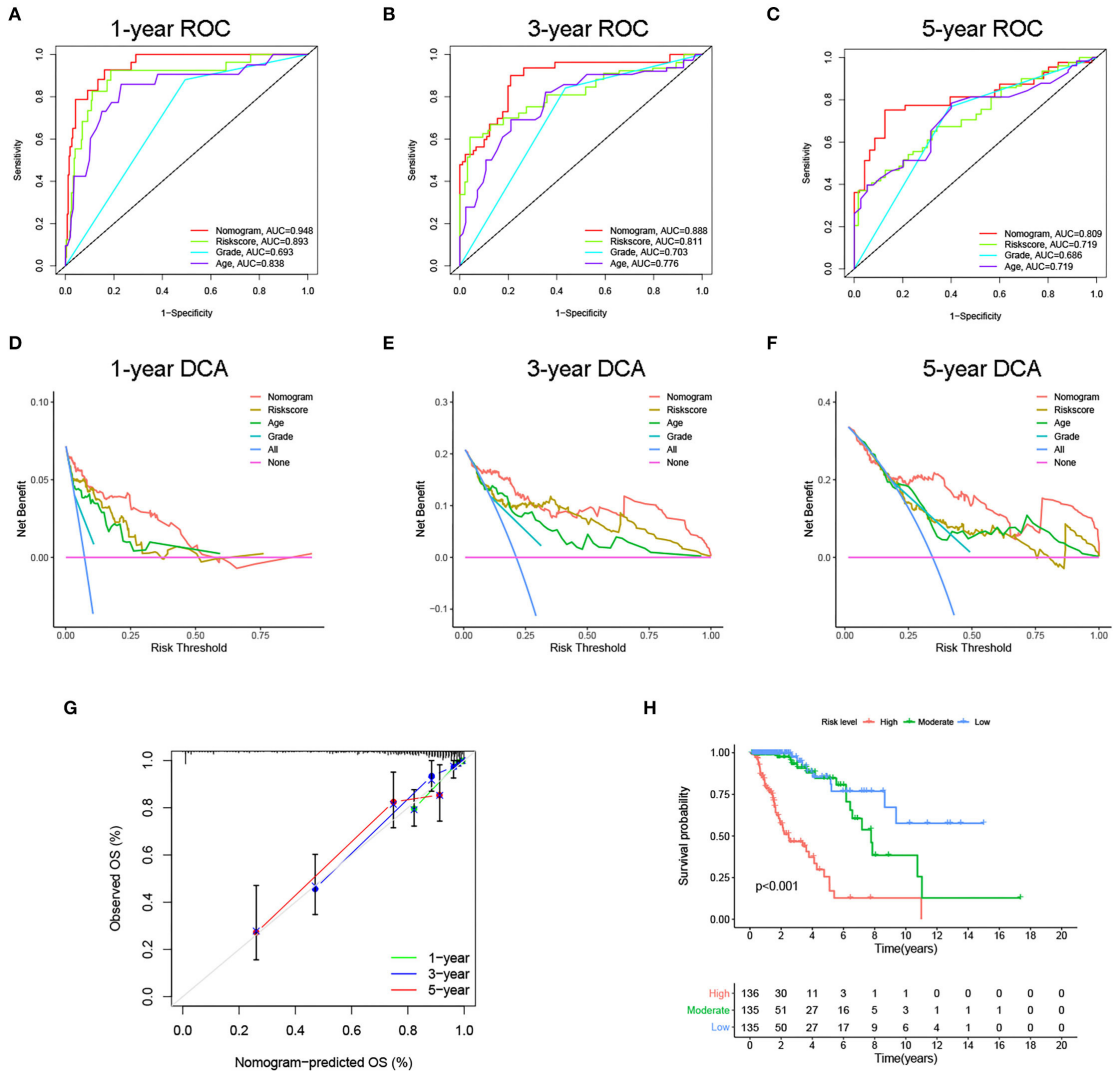
Internal evaluation of the nomogram model. **(A–C)** Time-dependent ROC curves, **(D–F)** decision curve analyses (DCAs) and **(G)** calibration curves for 1-, 3-, and 5-year overall survival (OS) prediction of the nomogram. **(H)** Kaplan–Meier curve of the three subgroups based on the nomogram.

Next, to identify independent prognostic indicators, univariate and multivariate Cox regression analyses were conducted. In TCGA cohort, outcomes revealed that grade, age, and risk score [hazard ratio (HR) = 2.387, 95% confidence interval (CI) = 1.647–3.457; *p* < 0.001] were independent prognostic indicators of LGG (Figures 5A,B). Interestingly, this risk signature also had independent prognostic significance in both the CGGA (HR = 2.848, 95% CI = 1.964**–**4.129; *p* < 0.001) and Gravendeel (HR = 4.148, 95% CI = 2.315**–**7.433; *p* < 0.001) cohorts (Figures 5C–F), further demonstrating that the ability of the risk signature to predict LGG survival was reliable and stable. We then incorporated the independent prognostic parameters of the TCGA cohort to build a nomogram model for individualized prognostic prediction of patients with LGG (Figure 6). Compared with independent prognostic factors, the tROC curves confirmed that the nomogram had the highest prediction accuracy, with AUCs of 0.948, 0.888, and 0.809 for 1-, 3-, and 5-year survival, respectively (Figures 7A–C). DCAs demonstrated that the nomogram was more beneficial than a single independent prognostic factor in predicting LGG outcomes (Figures 7D–F). Moreover, the model had a high C-index (0.869), further demonstrating its good predictive performance. We next plotted calibration curves for both the training and validation cohorts, and the results illustrated that the prediction probability through the nomogram was close to the actual observation (Figure 7G; Supplementary Figures 1A,B). The TCGA cohort was then divided into three subgroups based on model scores, and the Kaplan–Meier curve exhibited significant differences among groups (*p* < 0.001; Figure 7H). Similar results were observed in the CGGA cohort (Supplementary Figure 1C), however, in the Gravendeel cohort, the difference between the low- and moderate-risk groups was not significant, possibly due to its small sample size (Supplementary Figure 1D). Overall, these results suggested that the nomogram could be used as an effective risk stratification method.

### Functional Enrichment Analyses

**Figure 8:**
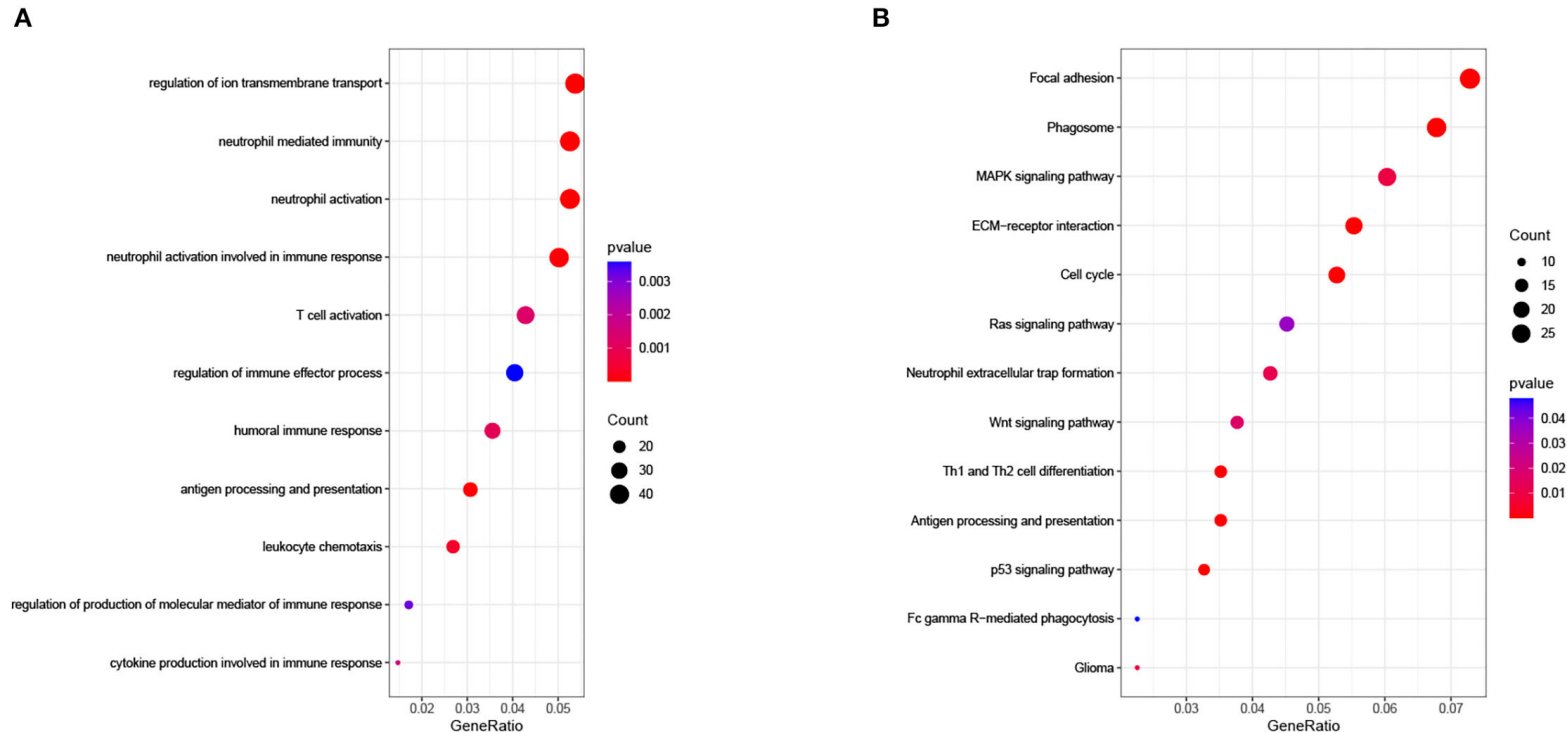
**(A)** Biological process of Gene Oncology (GO) analysis and **(B)** Kyoto Encyclopedia of Genes and Genomes (KEGG) pathway analysis in The Cancer Genome Atlas (TCGA) cohort.

To explore the related biological functions and pathways, functional enrichment analyses were carried out in the TCGA cohort. Between the two groups, 906 genes met the criteria and were defined as DEGs. Based on DEGs, the biological process (BP) results of GO analysis illustrated that the risk signature was closely linked to immune-related functions (Figure 8A). For KEGG analysis, the results suggested that DEGs were involved in extracellular matrix (ECM)-receptor interaction, phagosome, cell cycle, antigen processing and presentation, and some cancer pathways (Figure 8B).

### Relationship of the Risk Signature With Immunity and m6A

**Figure 9:**
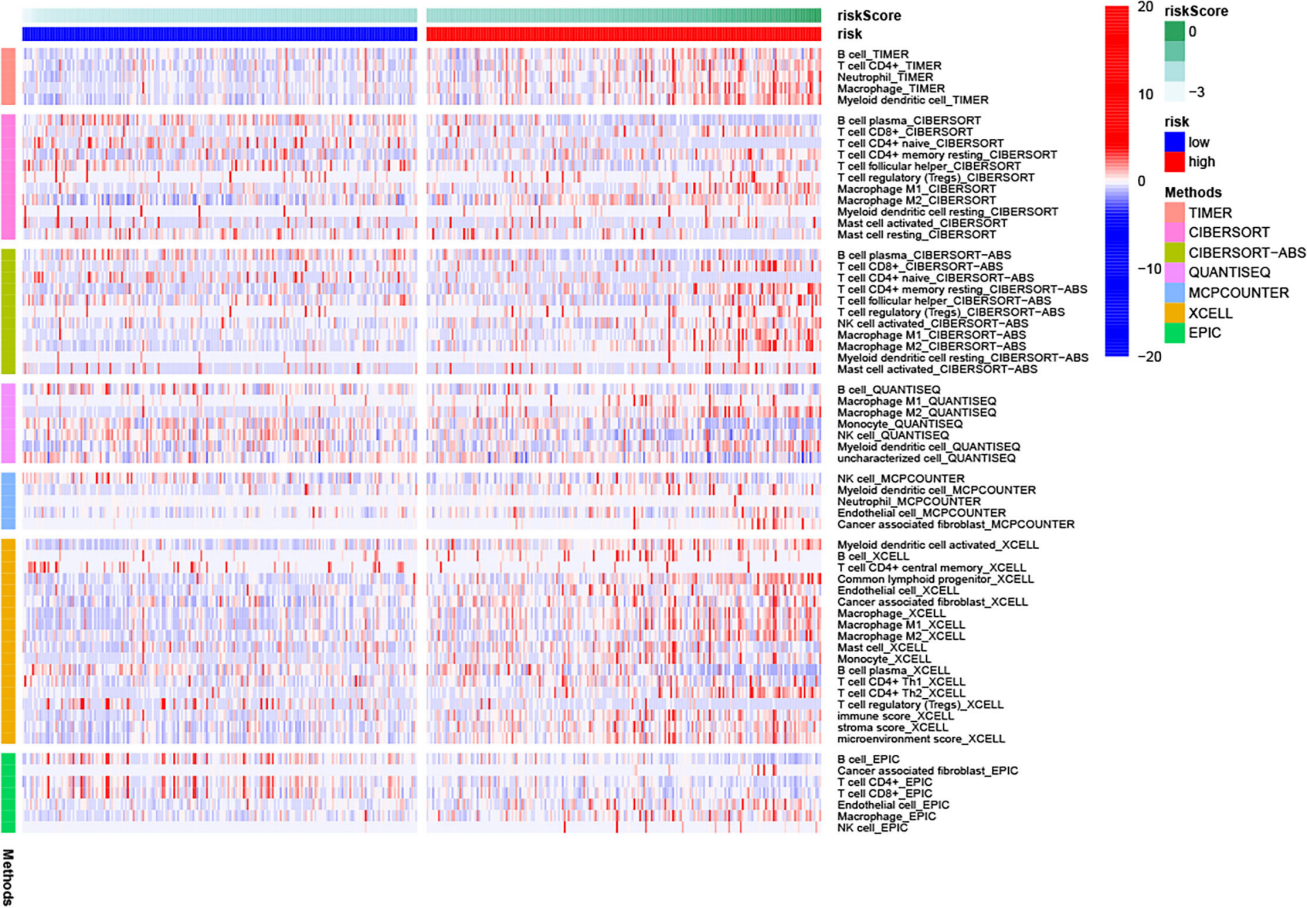
Immune cells and immune responses based on several algorithms.

**Figure 10:**
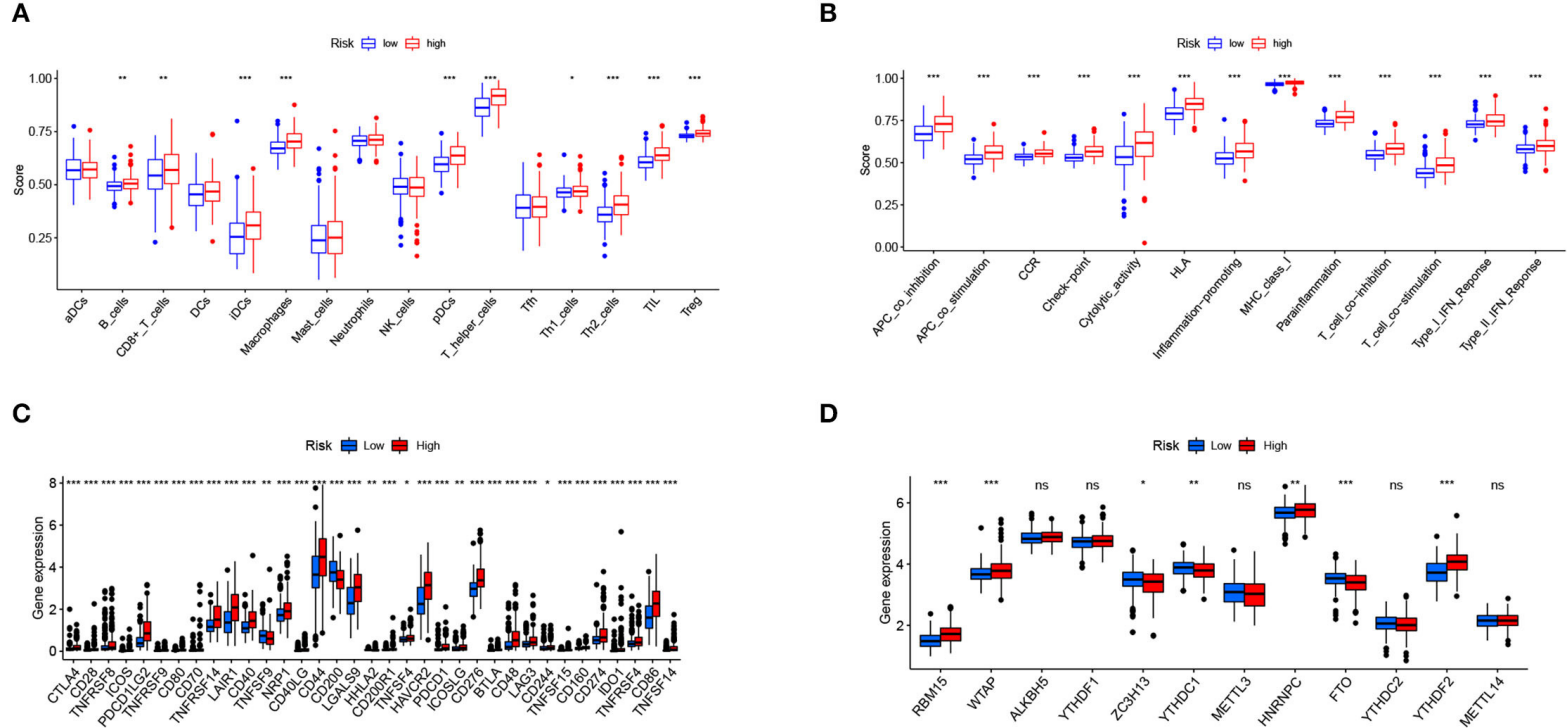
Relationship of the risk signature with immunity and m6A. **(A)** Scores of immune cell infiltration, **(B)** immune function activity, **(C)** immune checkpoint expression, and **(D)** m6A-related gene expression were compared between the two subgroups. * *p* < 0.05, ** *p* < 0.01, *** *p* < 0.001, ns, no significance.

Figure 9 shows the immune cells and immune responses that obviously differed in several algorithms. The ssGSEA results showed that the infiltration levels of B cells, CD8+ T cells, immature dendritic cells (iDCs), macrophages, plasmacytoid dendritic cells (pDCs), T helper cells, Th1 cells, Th2 cells, tumor-infiltrating lymphocyte (TIL) cells, and Regulatory T cell (Treg) cells were higher in the high-risk group than in the low-risk group (*p* < 0.05; Figure 10A). Meanwhile, all 13 immune-related pathways and functions exhibited higher activity in patients with high-risk scores (*p* < 0.001; Figure 10B). In addition, there were significant differences in the expression of many immune checkpoints. For example, in the high-risk group, the levels of CTLA4, PDCD1LG2 (PD-L2), PDCD1 (PD-1), LAG3, and CD274 (PD-L1) were much higher (*p* < 0.001; Figure 10C). Considering the key regulatory role of m6A-related genes in tumors, the relationship between the risk signature and m6A-related genes was analyzed. As shown in Figure 10D, we found that, in the high-risk group, the expression levels of RNA binding motif protein 15 (RBM15), Wilms tumor 1 associated protein (WTAP), heterogeneous nuclear ribonucleoprotein C (HNRNPC), and YTH N6-methyladenosine RNA binding protein 2 (YTHDF2) are higher (*p* < 0.01), whereas the expression levels of zinc finger CCCH-type containing 13 (ZC3H13), YTH domain containing 1 (YTHDC1), and fat mass and obesity associated (FTO) are lower than the low-risk group (*p* < 0.05).

## Discussion

Ferroptosis, a newly discovered form of iron-dependent programmed cell death (PCD), plays a vital role in malignant tumor biology that includes resistance to chemotherapy and tumor suppressor functions, which may be a promising strategy for cancer treatment (11, 14, 24). Hence, a comprehensive analysis of the prognostic value of ferroptosis-related lncRNAs in LGG is helpful to identify reliable prognostic markers and potential therapeutic targets. In this study, we identified 11 prognostic ferroptosis-related lncRNAs in LGG through three datasets (TCGA, CGGA, and Gravendeel), of which 8 were included to establish a risk signature. Through external validation, this risk signature exhibited a robust and accurate predictive capacity for survival in patients with LGG. The findings of our study also suggested that LGG types with worse prognosis, such as age > 40, grade III, IDH wild-type, and 1p19q non-codeletion, tended to have higher risk scores. In addition, the lncRNA signature-based nomogram we established had high AUC values (0.948, 0.888, and 0.809 for 1-, 3-, and 5-year OS, respectively) and C-index (0.869), which seemed to be superior to some nomograms previously constructed for LGG, such as that by Tu et al. (25) (AUC: 0.899, 0.860, and 0.806 for 1-, 3-, and 5-year OS, respectively; C-index: 0.817), Feng et al. (26) (C-index: 0.852), and Zhao et al. (27) (C-index: 0.777). Previous studies have shown that the ferroptosis-related lncRNA signature performs well in predicting the prognosis of various cancers, such as liver cancer, head, and neck squamous cell carcinoma, colon cancer, and lung adenocarcinoma (8, 28–30). Nevertheless, until now, there has been a lack of ferroptosis-related lncRNA signatures for LGG. This study is a useful complement to the prognostic indicators of LGG.

The risk signature of our study included 8 lncRNAs (CRNDE, LINC00844, FAM66C, TUBA3FP, SNHG8, HAR1A, LINC00641, and MYCNOS), and previous evidence has suggested that these lncRNAs are closely linked to the occurrence and development of cancer. For instance, the expression of the lncRNA CRNDE was significantly increased in gliomas, and high CRNDE expression was linked to a higher degree of malignancy and shorter OS time (31). Wang et al. (32) found that the upregulation of CRNDE enhanced the growth and migration of glioma cells, and the expression of CRNDE was regulated by mammalian target of rapamycin (mTOR) signaling. In the present study, CRNDE was primarily enriched in the high-risk group, and its expression was negatively associated with the prognosis of patients with LGG, which was consistent with previous findings. In prostate cancer, LINC00844 upregulates GSTP1 and promotes apoptosis by recruiting EBF1 (33). LINC00844 also has a significant prognostic value in gliomas, with a high expression suggesting a favorable prognosis (34). LINC00641 is a novel acute myeloid leukemia (AML)-related lncRNA whose knockdown prevents cell proliferation, invasion, and migration and promotes apoptosis by modulating the miR-378a/ZBTB20 axis in AML (35). Small nucleolar RNA host gene 8 (SNHG8) is an oncogenic factor involved in many types of cancer and is considered a promising target for cancer therapy (36). A previous study showed that high expression of lncRNA HAR1A was linked to better clinical outcomes for LGG, and upregulation of HAR1A helped to improve survival in patients who received chemotherapy and radiotherapy (37). This is consistent with our findings. With regard to MYCNOS, it has been reported to affect the growth of neuroblastoma cells by facilitating MYCN protein levels (38–40). However, until now, there have been few studies on the role and mechanism of ferroptosis-related lncRNAs in the prognosis of LGG. The potential regulatory role of these lncRNAs during the ferroptosis process needs to be further studied.

The exciting thing is that targeting ferroptosis exhibits a unique vulnerability for the treatment of some therapy-resistant tumors (41). Ferroptosis-based therapies have great potential in terms of the multiple resistance mechanisms caused by cellular plasticity switches, which can prevent therapeutic evasion and metastasis of malignancies from a variety of origins (42–44). In the present study, we found that DEGs between the two risk subgroups were closely related to multiple immune-related functions. The ssGSEA results showed higher levels of immune cell infiltration and more active immune-related functions in the high-risk group. In addition, several important immune checkpoints were expressed at higher levels in LGG patients with higher risk scores. To sum up, our data revealed that ferroptosis was linked to the immunity of LGG to some extent. Recent studies suggest that ferroptosis inducers combined with immune checkpoint inhibitors may be an effective method to enhance antitumor effects (45, 46). Therefore, this combination therapy may be a promising treatment approach for high-risk patients with LGG based on our signature. In the KEGG analysis, the risk signature was associated with ECM-receptor interaction and focal adhesion, suggesting that the two glioma subgroups may differ in their ability to invade and migrate (47, 48). In addition, the DEGs were significantly enriched in several cancer-related pathways, such as mitogen-activated protein kinase (MAPK), p53, and Wnt signaling pathways. The abnormality of the m6A gene is closely linked to the occurrence and development of glioma (49). In our study, the expression levels of RBM15, WTAP, HNRNPC, YTHDF2, ZC3H13, YTHDC1, and FTO were significantly different in the two subgroups. These findings further shed light on the reasons for the differences in survival time between the two subgroups.

However, there are some limitations to this study. First, the public databases included in this study are deficient to varying degrees, lacking some key clinical parameters, such as tumor resection degree and preoperative status of patients. Second, the relatively small sample size of the Gravendeel cohort may affect the accuracy of the partial validation results. Finally, further experiments are needed to elucidate the underlying mechanisms related to the risk signature in LGG. In summary, the ferroptosis-related lncRNA signature exhibits good performance in predicting the prognosis of LGG. Ferroptosis-related lncRNAs may influence the prognosis of LGG in part by modulating immune-related functions. Our study may provide useful insight into the treatment of patients with LGG.

## Data Availability Statement

The original contributions presented in the study are included in the article/Supplementary Material, further inquiries can be directed to the corresponding authors.

## Ethics Statement

Ethical review and approval was not required for the study on human participants in accordance with the local legislation and institutional requirements. Written informed consent from the patients/participants' legal guardian/next of kin was not required to participate in this study in accordance with the national legislation and the institutional requirements.

## Author Contributions

Q-RH and J-WL participated in data analysis and drafted the manuscript. PY, QJ, and F-ZG were responsible for data collection, chart making, and assisted in data analysis. Y-NZ and L-GM performed study concept, design, and revised the manuscript. All authors read and approved the final manuscript.

## Conflict of Interest

The authors declare that the research was conducted in the absence of any commercial or financial relationships that could be construed as a potential conflict of interest.

## Publisher's Note

All claims expressed in this article are solely those of the authors and do not necessarily represent those of their affiliated organizations, or those of the publisher, the editors and the reviewers. Any product that may be evaluated in this article, or claim that may be made by its manufacturer, is not guaranteed or endorsed by the publisher.
