# Supplementary figures and images for: Establishment and Validation of a Ferroptosis-Related lncRNA Signature for Prognosis Prediction in Lower-Grade Glioma
Source: Front Neurol. 2022 Jun 27;13:861438. doi: 10.3389/fneur.2022.861438 (PMC9271629; doi:10.3389/fneur.2022.861438)

A

CGGA

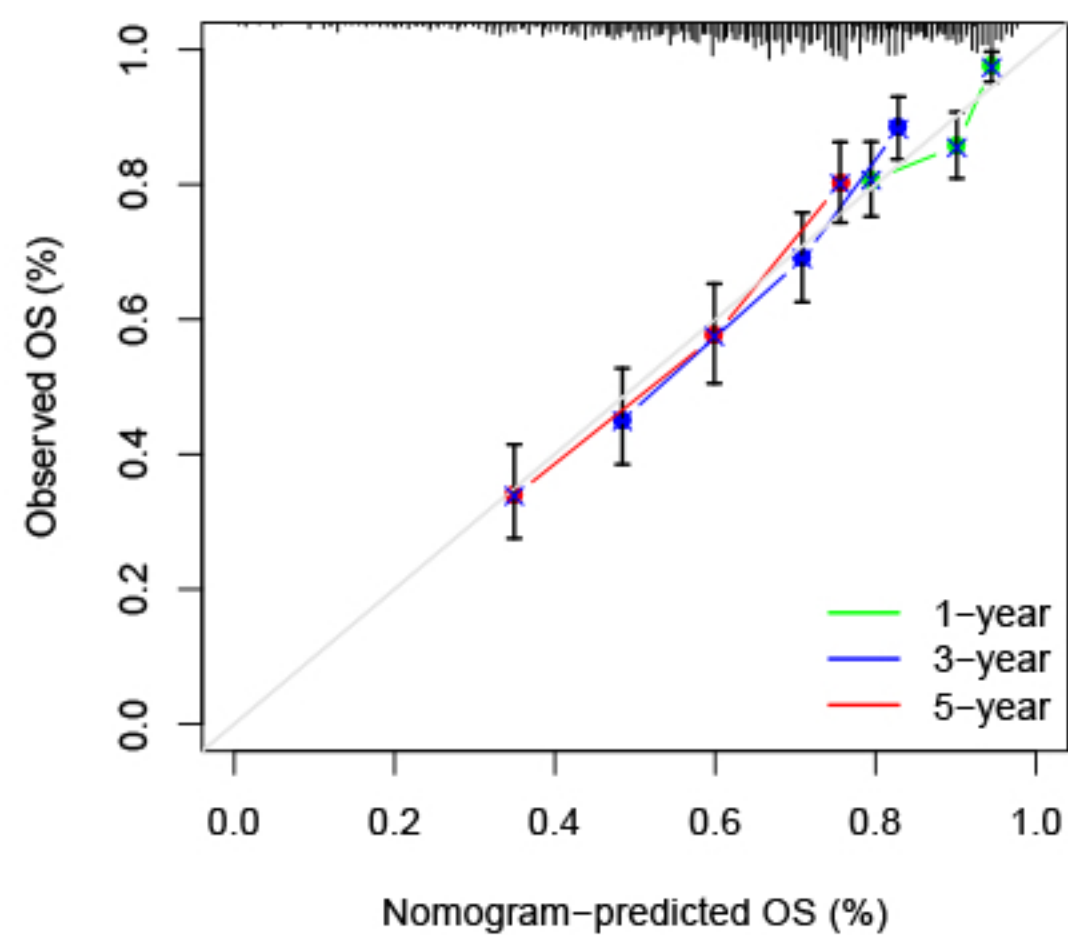

B

Gravendeel

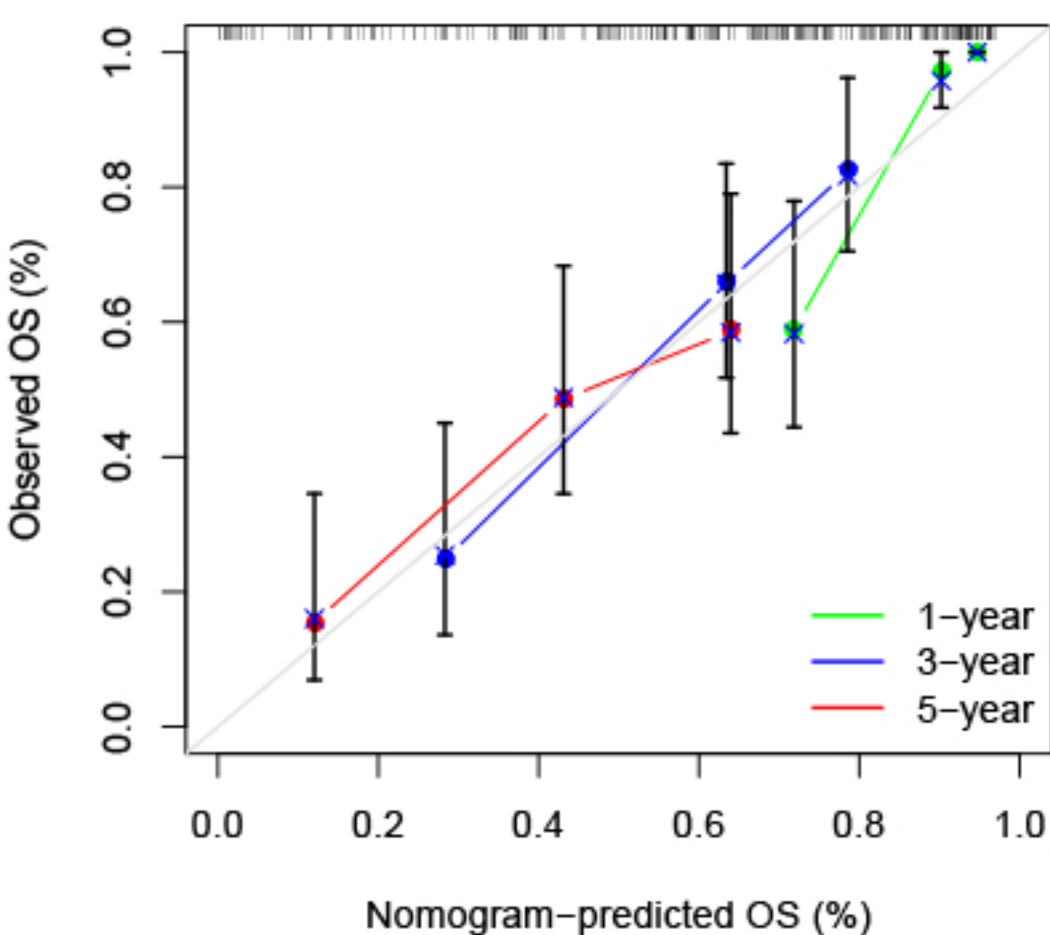

C

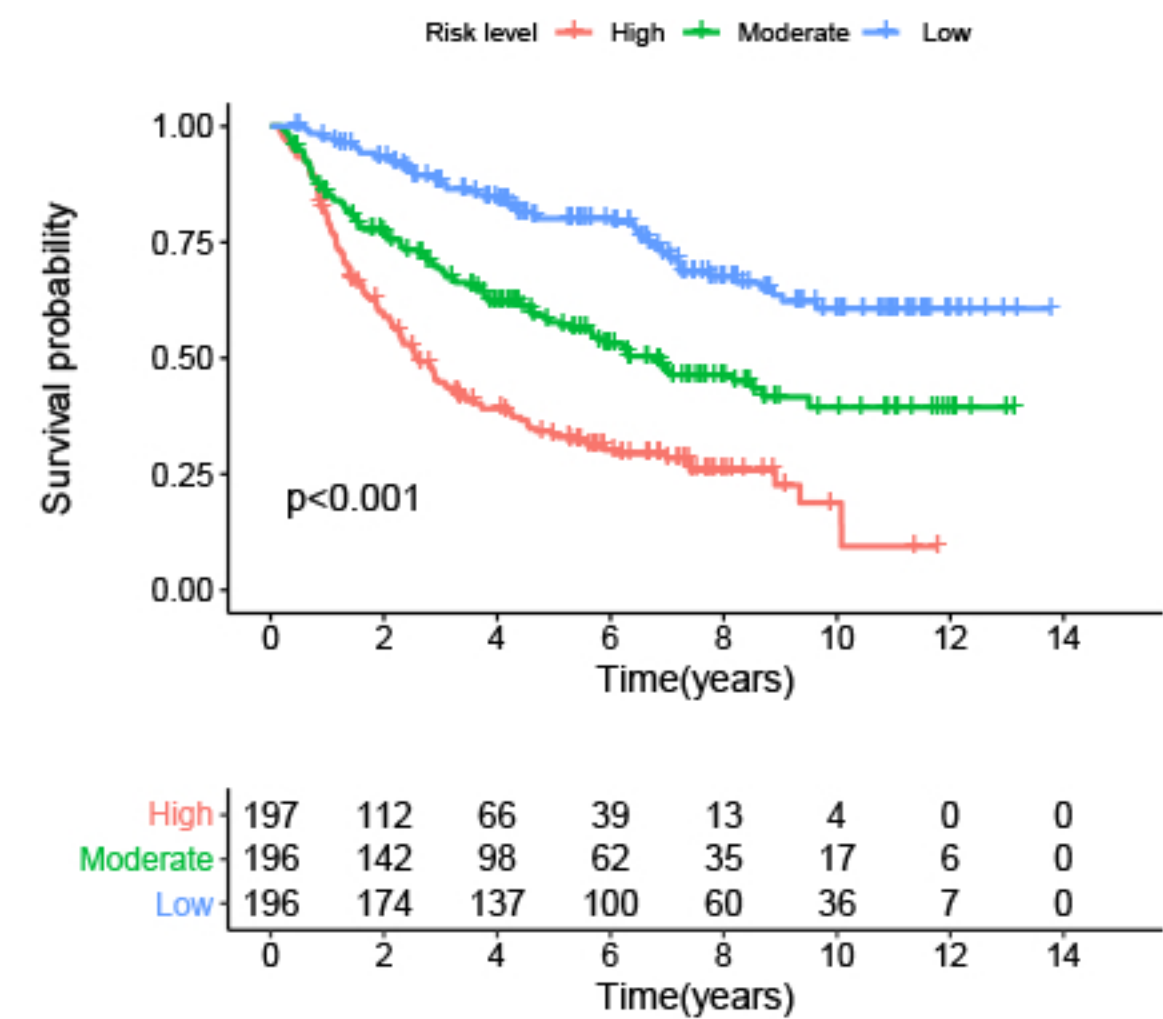

D

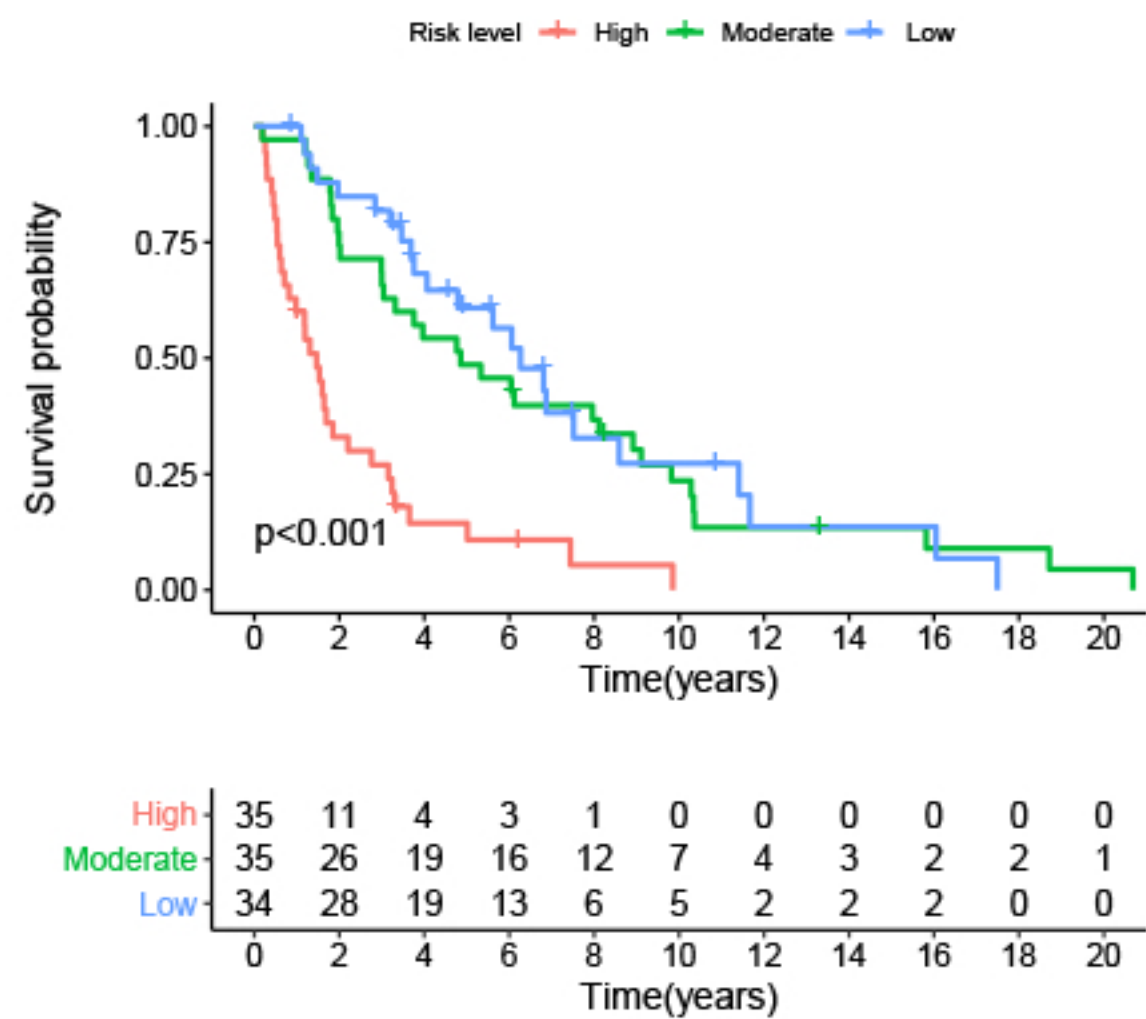

Supplement: Supplementary file 1 [file Image_1.pdf]

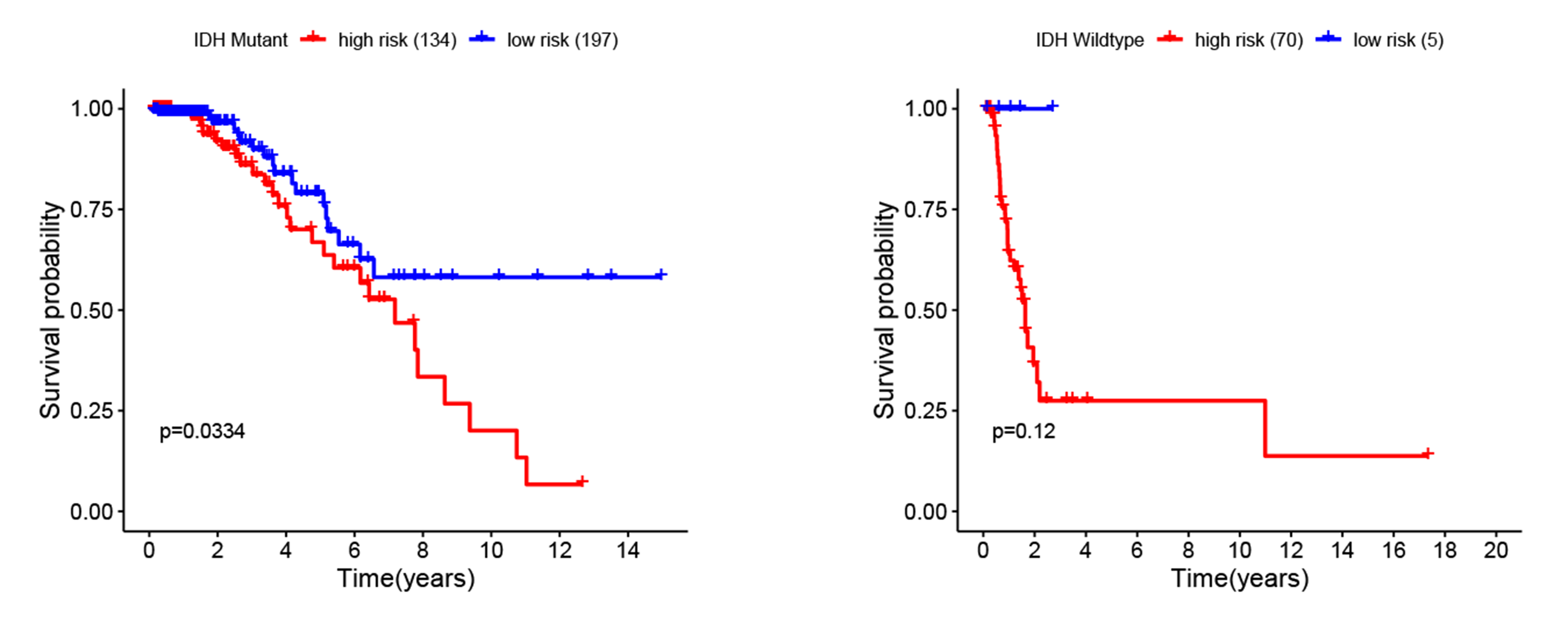

Supplement: Supplementary file 2 [file Image_1.TIF]
